# An Active Component of *Achyranthes bidentata* Polypeptides Provides Neuroprotection through Inhibition of Mitochondrial-Dependent Apoptotic Pathway in Cultured Neurons and in Animal Models of Cerebral Ischemia

**DOI:** 10.1371/journal.pone.0109923

**Published:** 2014-10-15

**Authors:** Shu Yu, Caiping Wang, Qiong Cheng, Hui Xu, Shibo Zhang, Lu Li, Qi Zhang, Xiaosong Gu, Fei Ding

**Affiliations:** Jiangsu Key Laboratory of Neuroregeneration, Collaborative Innovation Center of Neuroregeneration, Nantong University, Nantong, Jiangsu, China; University of Louisville, United States of America

## Abstract

An active component has been isolated by reverse-phase high performance liquid chromatography (HPLC) from *Achyranthes bidentata* Blume polypeptides that are extracted from *Achyranthes bidentata* Blume, a Chinese medicinal herb. The active component is called ABPPk based on the order of HPLC elution. In this study, we used *in vitro* and *in vivo* experimental models of cerebral ischemia to investigate the possible neuroprotective effect of ABPPk. ABPPk treatment promoted neuronal survival and inhibited neuronal apoptosis in primary cortical neurons exposed to oxygen and glucose deprivation and in rats subjected to transient middle cerebral artery occlusion. The role of ABPPk in protection against ischemia-induced neuronal damage might be mediated by mitochondrial-dependent pathways, including modulation of apoptosis-related gene expression, regulation of mitochondrial dysfunction through restoring mitochondrial membrane potential, reducing release of mitochondrial apoptogenic factors, and inhibiting intracellular ROS production. The neuroprotective effect of ABPPk may suggest the possible use of this agent in the treatment and prevention of cerebral ischemic stroke.

## Introduction

Cerebral ischemic stroke is a major cause of death and disability worldwide. This neurological disease is caused by an obstruction of blood flow to the brain, which triggers the onset of ischemic cascades, including energy failure, excitotoxicity, oxidative stress, inflammation, and apoptosis. To understand the molecular mechanisms of neuronal damage during ischemic stroke, and develop neuroprotective drugs/agents against ischemic injury, oxygen and glucose deprivation (OGD) and middle cerebral artery occlusion (MCAO) are extensively used as *in vitro* and *in vivo* models of cerebral ischemia, respectively.

*Achyranthes bidentata* Blume (Amaranthaceae family), a traditional Chinese medicinal herb, has a range of pharmacological properties [1]. The aqueous extract of *Achyranthes bidentata* Blume has been shown to promote peripheral nerve regeneration in rabbits with crush injury to the common peroneal nerve [2]. From the aqueous extract of *Achyranthes bidentat*a Blume, we isolated *Achyranthes bidentata* polypeptides (ABPP), which attenuated the glutamate-induced apoptosis in primary hippocampal neurons, supported recovery from experimental cerebral ischemia *in vivo* [3]–[5], and promoted peripheral nerve regeneration in rodents with sciatic nerve crush through stimulating release of growth factors [6], [7]. To improve the pharmaceutical value of ABPP, we used DEAE anion exchange high-performance liquid chromatography (HPLC) to separate the components of ABPP, one of which (referred to as ABPP-E4) exhibited protective effects against serum deprivation-induced neuronal apoptosis in SH-SY5Y cells [8]. On the other hand, we used reverse phase HPLC to separate several active components from ABPP, and one of these components, called ABPPk according to the elution order on C18 reverse phase column, showed the best neuroactivity among all components.

This study was designed to investigate the potential neuroprotective effects of ABPPk by using the aforementioned *in vitro* and *in vivo* models of cerebral ischemia. We found that ABPPk inhibited neuronal insult in primary cortical neurons exposed to OGD and in rats subjected to MCAO-induced brain ischemia. We also explored the mitochondrial regulation mechanisms responsible for neuroprotection of ABPPk.

## Results

### ABPPk reduced cell viability loss in primary cortical neurons exposed to OGD and diminished brain infarct formation in rats subjected to MCAO

**Figure 1:**
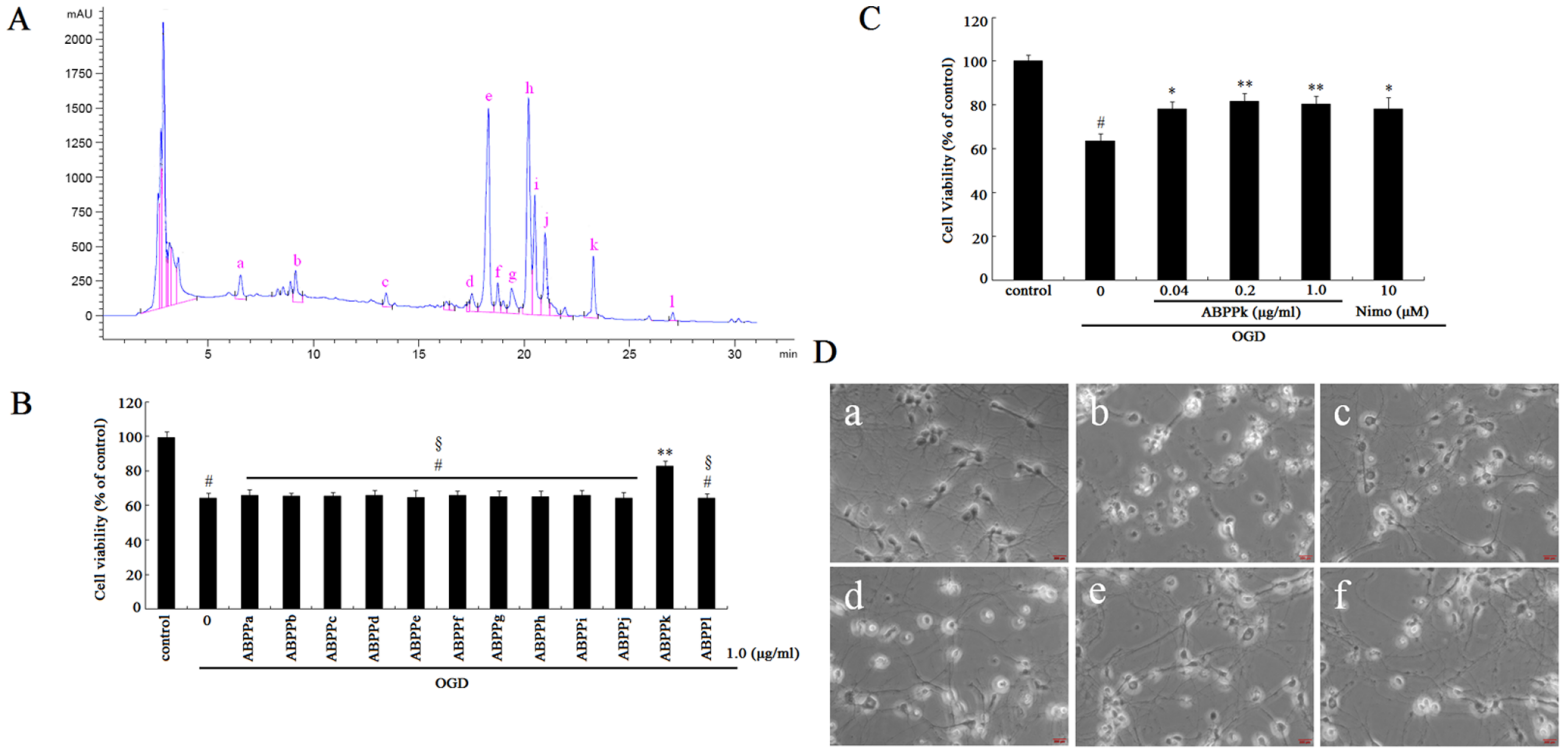
Neuroprective effects of ABPPk in OGD model. (A) Representative HPLC chromatogram of *Achyranthes bidentata* Blume polypeptides (ABPP), recorded at 220 nm. (B) MTT assay was used to measure cell viability. Histogram showing cell viability of primary cortical neurons after different treatments designed as follows: no treatment (control), exposure to OGD alone, exposed to OGD followed by treatment with 1.0 µg/ml of ABPPa-ABPPl, respectively. ^#^ *P*<0.01 versus control, **P<0.01 versus OGD alone, ^§^ *P*<0.01 versus OGD plus treatment with 1.0 µg/ml of ABPPk. (C) MTT assay was used to measure cell viability. Histogram showing cell viability of primary cortical neurons that had been exposed to OGD alone, exposed to OGD and then treated with 0.04, 0.2, or 1.0 µg/ml of ABPPk or with 10 µM nimodipine (Nimo), respectively. ^#^ *P*<0.01 versus control (cells without any treatment), *P<0.05 and **P<0.01 versus OGD alone. (D) Phase-contrast micrograph showing the morphological changes in primary cortical neurons without any treatment (a, control), exposed to OGD alone (b), exposed to OGD and then treated with 0.04 (c), 0.2 (d), or 1.0 (e) µg/ml of ABPPk, or 10 µM nimodipine (Nimo, f), respectively. Scale bar, 200 µm.

ABPP was separated by reverse-phase HPLC with a photodiode array (PDA) detector to obtain 12 different HPLC fractions. These ABPP components were labeled as ABPPa, b, c, d, e, f, g, h, i, j, k, l, respectively, according to the elution order. A representative chromatogram was recorded at 220 nm (Fig. 1A). 3-(4,5-dimethylthiazol-2-yl)-2,5-diphenyl tetrazolium bromide (MTT) assay was used to determine cell survival of primary cortical neurons after cell treatments. Exposure to OGD decreased cell viability of primary cortical neurons as compared to control (no treatment). An equal dose (1.0 µg/ml) of 12 ABPP components was used to treat OGD-injured primary cortical neurons. MTT assay compared the effects of 12 ABPP components on cell viability, and confirmed that only ABPPk treatment significantly attenuated OGD-induced decrease in cell viability (Fig. 1B). Furthermore, the effect of ABPPk (dose range, 0.04–1.0 µg/ml) displayed a dose-dependent pattern, and 0.2 or 1.0 µg/ml of ABPPk yielded the neuroprotective effect similar to that of 10 µM nimodipine, a calcium-channel blocker, which was chosen as positive control in this study (Fig. 1C). It should be mentioned that treatment with ABPPk alone caused no cytotoxicity on primary cortical neurons (data not shown).

After exposure to OGD, primary cortical neurons showed morphological alterations, such as neurite disappearance and vacuolus emergence around the cell body, but ABPPk treatment enabled OGD-injured neurons to reestablish the normal cell morphology (Fig. 1D).

**Figure 2:**
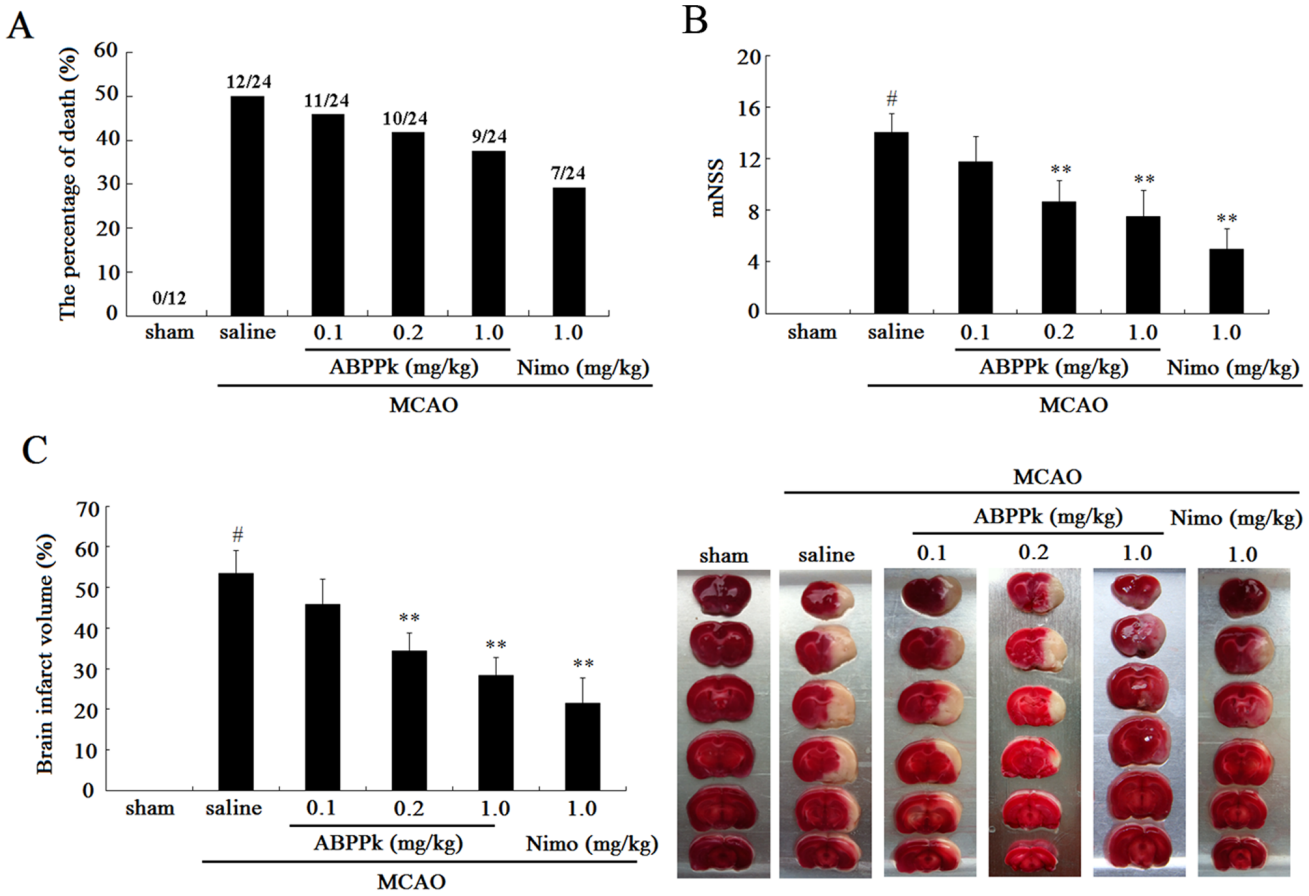
Neuroprective effects of ABPPk in MCAO model. (A, B and C) Histograms respectively comparing the percentage of animal death (mortality) (A), the mNSS (B) and the brain infarct volume (C) among sham-MCAO rats, MCAO rats receiving administration of saline, 0.1, 0.2, or 1.0 mg/kg of ABPPk, or 1.0 mg/kg nimodipine (Nimo), respectively. ^#^ *P*<0.01 versus sham-MCAO rats, **P<0.01 versus MCAO rats receiving saline treatment. The numerator and denominator of the fraction on the top of bars represent the number of dead and total animals respectively (A). Also shown in inset of (C) is a representative TTC staining image of rat brain slices.

To determine the neuroprotective action of ABPPk *in vivo*, rats were subjected to MCAO-induced ischemia. At the end of MCAO and the start of reperfusion, rats were intravenously (iv) administered with 1 ml of 0.1, 0.2, or 1.0 mg/kg ABPPk, 1 ml of 1.0 mg/kg nimodipine, and 1 ml of saline (vehicle), respectively. The neurological deficits were then evaluated by measuring the percentage of animal death (mortality), modified neurological severity score (mNSS), and brain infarct volume. Each of 3 indexes was significantly higher in MCAO rats treated with saline (control) than in sham-MCAO rats. ABPPk treatment reversed MCAO-induced increase in 3 indexes in a dose-dependent manner with significant differences in mNSS and brain infarct volume occurring between treatment with 0.2 or 1.0 mg/kg of ABPPk and treatment with saline. However, the reversing effect of all doses of ABPPk was slightly lower than that of 1.0 mg/kg nimodipine (Fig. 2).

### ABPPk protected primary cortical neurons and rat brain against ischemia-induced neuronal apoptosis

**Figure 3:**
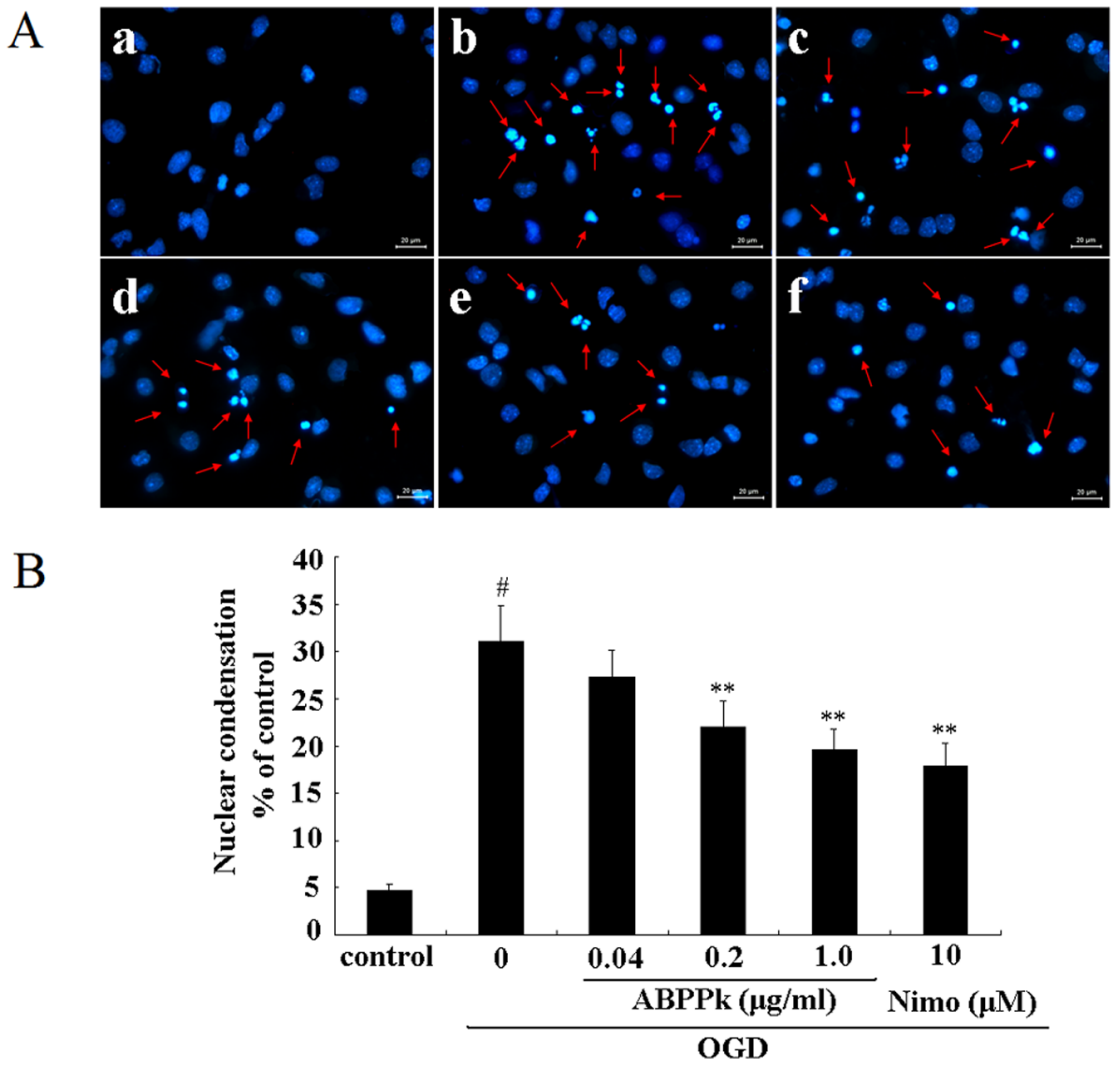
The effects of ABPPk on neuronal apoptosis in OGD model. (A) Representative fluorescence micrographs of Hoechst 33342 staining of primary cortical neurons without any treatment (a, control), exposed to OGD alone (b), exposed to OGD and then treated with 0.04 (c), 0.2 (d), or 1.0 (e) µg/ml of ABPPk, or 10 µM nimodipine (Nimo, f), respectively. The apoptotic cells were marked by arrows. Scale bar, 20 µm. (B) The resulting histogram showing the percentage of apoptotic cells (featured by nuclear condensation) in cell population after different cell treatments as indicated. ^#^ *P*<0.01 versus control, **P<0.01 versus OGD alone.

**Figure 4:**
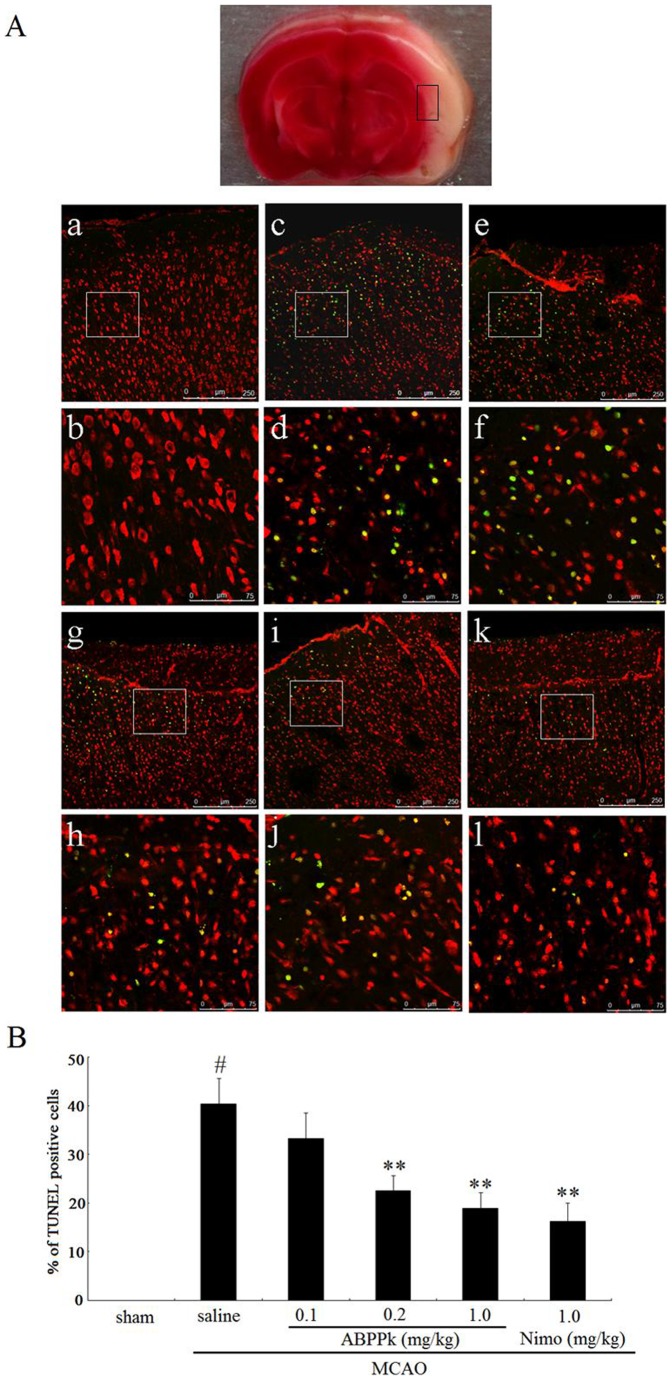
The effects of ABPPk on neuronal apoptosis in MCAO model. (A) Representative fluorescence micrographs of TUNEL staining of brain slices from sham-MCAO rats (a), MCAO rats receiving administration of saline (c), 0.1 (e), 0.2 (g), or 1.0 (i) mg/kg of ABPPk, or 1.0 mg/kg nimodipine (Nimo, k), respectively. The images (b, d, f, h, j, l) are the higher magnifications of the boxed areas in the images (a, c, e, g, i, k), respectively. Scale bar, 250 µm for (a, c, e, g, i, k) and 75 µm for (b, d, f, h, j, l). Apoptotic (TUNEL-positive) cells are detected as localized bright green cells in a red background: red color indicates normal cells stained with PI and green color indicates apoptotic cells stained with fluorescein-12-dUTP. Also shown (top) is the brain slice image with the anatomical position of ischemic penumbra region being labeled with a box. (B) The resulting histogram showing the percentage of apoptotic cells (TUNEL-positive) in cell population in brain slices of rats after treatments as indicated. ^#^ *P*<0.01 versus sham-MCAO rats, **P<0.01 versus MCAO rats receiving saline administration.

Hoechst 33342 staining indicated that following OGD, about 31% of primary cortical neurons exhibited apoptotic changes, such as chromatin condensation, nuclear shrinkage, and/or formation of apoptotic bodies, while ABPPk treatment reduced OGD-induced increase in the number of apoptotic cells (Fig. 3). Terminal deoxynucleotidyl transferase (TdT)-mediated dUTP-biotin nick end labeling (TUNEL) assay showed that MCAO induced a remarkable increase in the number of apoptotic (TUNEL-positive) cells, while ABPPk treatment significantly decreased MCAO-induced increase in the number of apoptotic (TUNEL-positive) cells (Fig. 4).

### ABPPk inhibited caspase-3 activation in primary cortical neurons and in rat brain after ischemic insult

Western blot analysis was applied to determine the protein expression of procaspase-3 and cleaved caspase-3 in the cell and tissue sample after ischemic insult.

**Figure 5:**
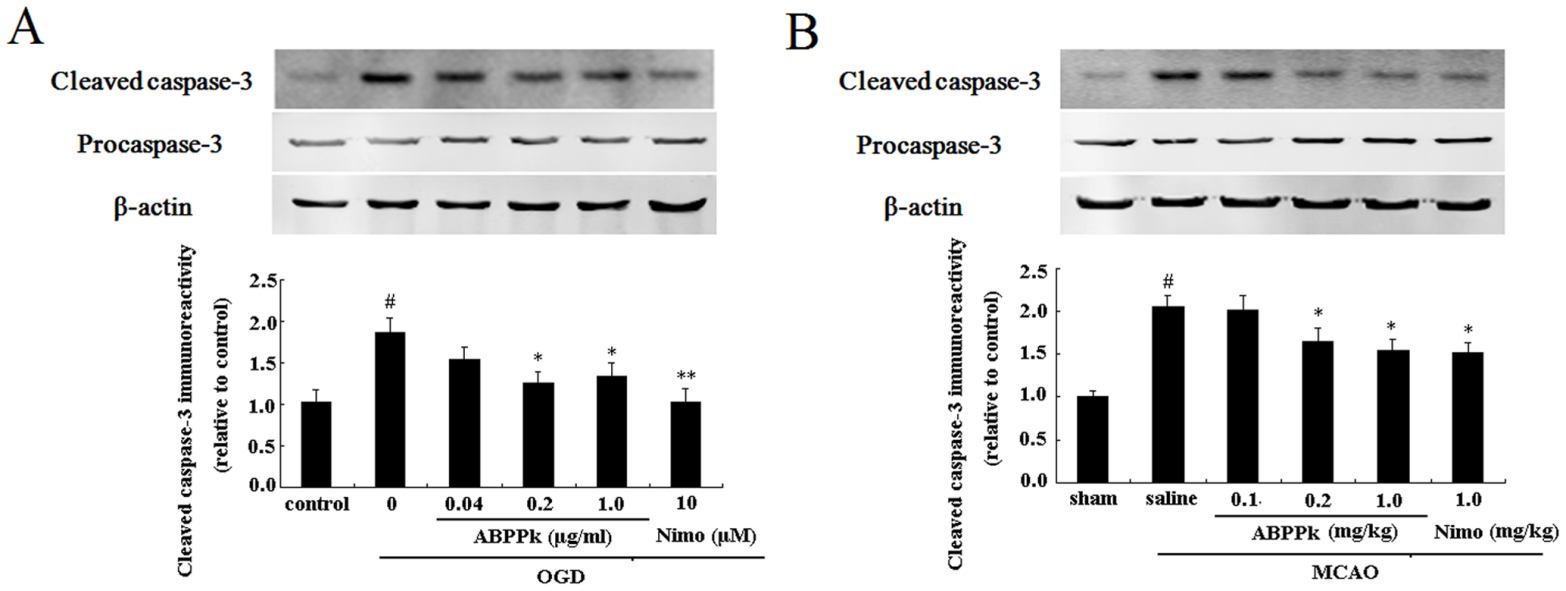
ABPPk-induced inhibition of caspase-3 activation in OGD and MCAO models. (A) Representative Western blot image (the upper) and the resulting histogram (the lower) showing the expression of cleaved caspase-3 in primary cortical neurons without any treatment (control), exposed to OGD alone, exposed to OGD and then treated with 0.04, 0.2, or 1.0 µg/ml of ABPPk or 10 µM nimodipine (Nimo), respectively. ^#^ *P*<0.01 versus control, *P<0.05 and **P<0.01 versus OGD alone. (B) Representative Western blot image (the upper) and the resulting histogram (the lower) showing the expression of cleaved caspase-3 in brain tissues of sham-MCAO rats, MCAO rats receiving administration of saline (vehicle), 0.1, 0.2, or 1.0 mg/kg of ABPPk, or 1.0 mg/kg nimodipine (Nimo). ^#^ *P*<0.01 versus sham-MCAO rats, *P<0.05 versus MCAO rats receiving saline administration. β-actin served as loading control.

It is known that ischemic injury triggers proteolytic processing of procaspase-3, resulting in the formation of a 17 kD subunit of the cleaved caspase-3 (active caspase-3). In this study, the expression of cleaved caspase-3 significantly increased in OGD-injured primary cortical neurons as compared to control cells while the expression of procaspase-3 was slightly decreased, and ABPPk treatment significantly antagonized OGD-induced upregulation of cleaved caspase-3 in primary cortical neurons in a dose-dependent manner (Fig. 5A). Similarly, a remarkable increase in cleaved caspase-3 expression was observed in the brain tissue of MCAO rats, and ABPPk treatment also significantly alleviated MCAO-induced upregulation of cleaved caspase-3 in the brain tissue in a dose-dependent manner (Fig. 5B).

### ABPPk reversed OGD-induced reduction of mitochondrial membrane potential in primary cortical neurons

**Figure 6:**
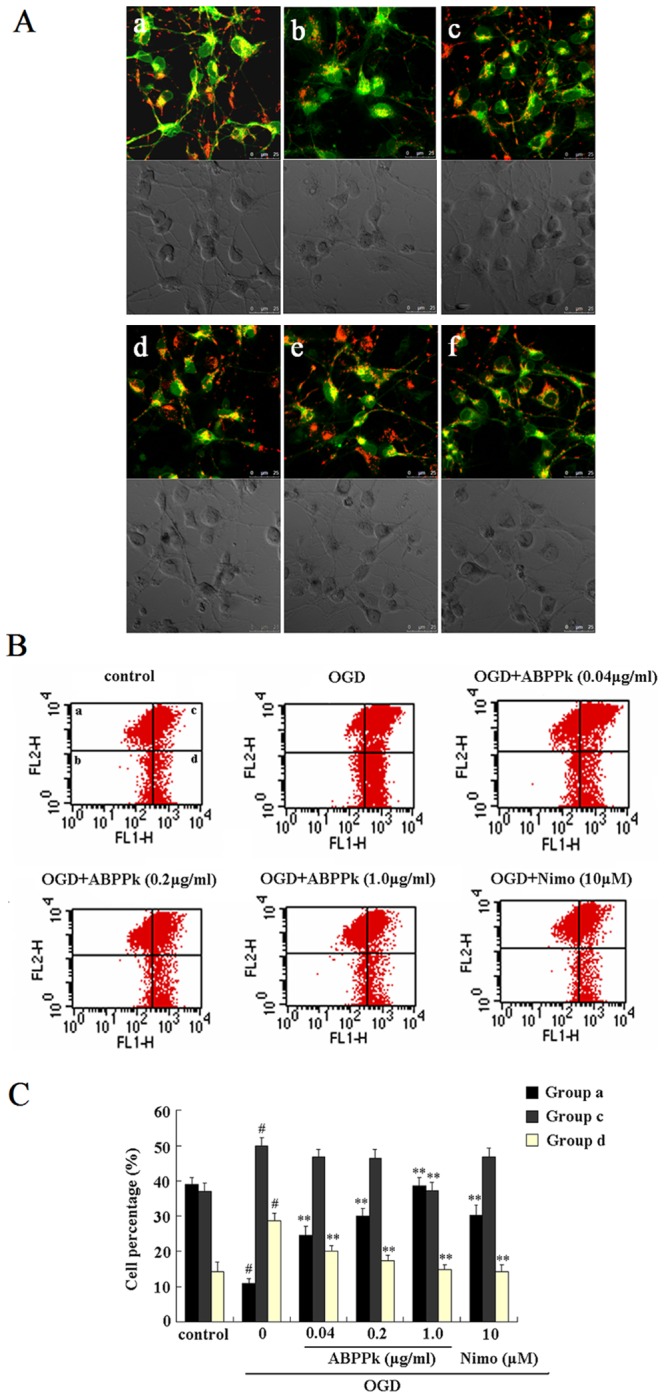
ABPPk-induced recovery of mitochondrial membrane potential in OGD model. (A) Representative fluorescent micrographs (a–f, top) of JC-1 staining and differential interference contrast (DIC) images (a–f, bottom) for comparing the mitochondrial membrane potential of primary cortical neurons without any treatment (a, control), exposed to OGD alone (b), exposed to OGD and then treated with 0.04 (c), 0.2 (d), or 1.0 (e) µg/ml of ABPPk, or 10 µM nimodipine (Nimo, f), respectively. Scale bar, 25 µm. (B) Flow cytometry with JC-1 staining for measuring the mitochondrial membrane potential of primary cortical neurons after different treatment as indicated. The JC-1 stained primary cortical neurons, which had been subjected to different treatments as indicated, were divided into 4 subpopulations (a, b, c, d), representing mitochondrial polarization or depolarization, respectively. (C) Histogram showing the percentage of 3 subpopulations (a, c and d). ^#^ *P*<0.01 versus control, **P<0.01 versus OGD alone.

A fall in mitochondrial membrane potential (ΔΨ_M_) is one of the earliest events in apoptosis [9]. Therefore, we examined the effect of ABPPk on ΔΨ_M_ of primary cortical neurons using 5,5′,6,6′-tetrachloro-1,1′,3,3′-tetraethyl-benzimidazol- carbocyanine iodide (JC-1), a mitochondrial-specific fluorescent probe. JC-1 staining of mitochondria caused a typical distribution of red and green fluorescent signals in primary cortical neurons without any treatment (control). After exposure to OGD, the ΔΨ_M_ in primary cortical neurons was significantly decreased, as indicated by an increase in green fluorescence and a decrease in red fluorescence. Treatment with ABPPk as well as nimodipine protected primary cortical neurons against OGD-induced decrease in ΔΨ_M_, or increase in mitochondrial depolarization (Fig. 6A).

Flow cytometry with JC-1 staining was used to confirm that ABPPk treatment prevented OGD-induced mitochondrial dysfunction in primary cortical neurons. The stained cells could be classified into four subpopulations: group A, those with high red and low green fluorescence representing normal mitochondrial polarization; group B, those with low red and low green fluorescence representing dead cells or debris; group C, those with high red and high green fluorescence representing normal mitochondrial polarization and cells with mitochondrial depolarization; and group D, those with low red and high green fluorescence representing mitochondrial depolarization. According to flow cytometric analysis, OGD decreased the ratio of red to green fluorescence (the ratio of mitochondrial polarization to mitochondrial depolarization) in JC-1 stained primary cortical neurons, but ABPPk treatment increased the cell population with normal mitochondrial polarization and decreased the cell population with mitochondrial depolarization in a dose-dependent manner. In other words, ABPPk could recover OGD-induced mitochondrial dysfunction in primary cortical neurons (Fig. 6B and 6C).

### ABPPk affected protein expression of Bcl-2, Bcl-xL, and Bax in primary cortical neurons exposed to OGD

**Figure 7:**
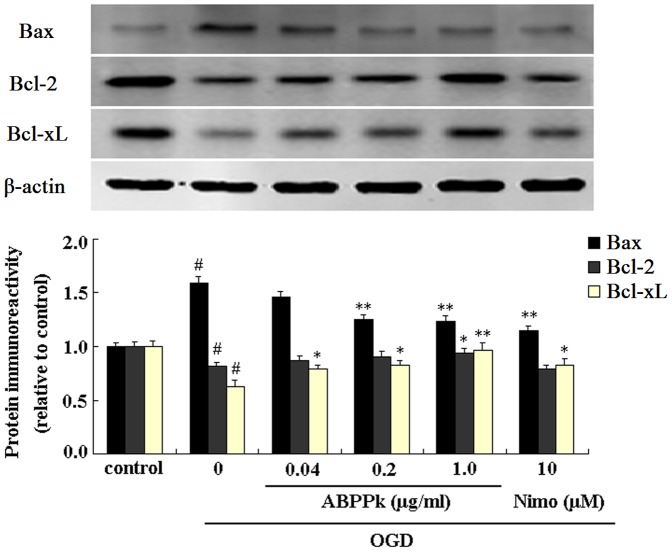
The effects of ABPPk on Bcl-2, Bcl-xL and Bax expression in OGD model. Representative Western blot image and the resulting histogram showing the protein expression of Bcl-2, Bcl-xL and Bax in primary cortical neurons without any treatment (control), exposed to OGD alone, exposed to OGD and then treated with 0.04, 0.2, or 1.0 µg/ml of ABPPk or 10 µM nimodipine (Nimo), respectively. ^#^ *P*<0.01 versus control, *P<0.05 and **P<0.01 versus OGD alone. β-actin served as loading control.

The intrinsic mitochondrial pathway of ischemia-induced apoptosis is mainly regulated by caspases and Bcl-2 family members [10], [11]. Bcl-2 family includes anti-apoptotic members (such as Bcl-2, Bcl-xL) and pro-apoptotic members (such as Bax, Bad, Bak, Bik, and Bcl-xs) [12]. In this study, we determined the changes in the protein expression of Bcl-2, Bcl-xL, and Bax using Western blot analysis. Exposure to OGD reduced the expression of Bcl-2 and Bcl-xL, but enhanced the expression of Bax. ABPPk treatment, in a dose-dependent manner, antagonized OGD-induced down-regulation of Bcl-2 and Bcl-xL and OGD-induced up-regulation of Bax, and significant reversing effects of ABPPk were achieved by 3 doses (0.04, 0.2 and 1.0 µg/ml) for Bcl-xL expression, 1 dose (1.0 µg/ml) for Bcl-2 expression, and 2 doses (0.2 and 1.0 µg/ml) for Bax expression (Fig. 7).

### ABPPk suppressed release of cytochrome c and apoptosis inducing factor (AIF) in primary cortical neurons exposed to OGD

**Figure 8:**
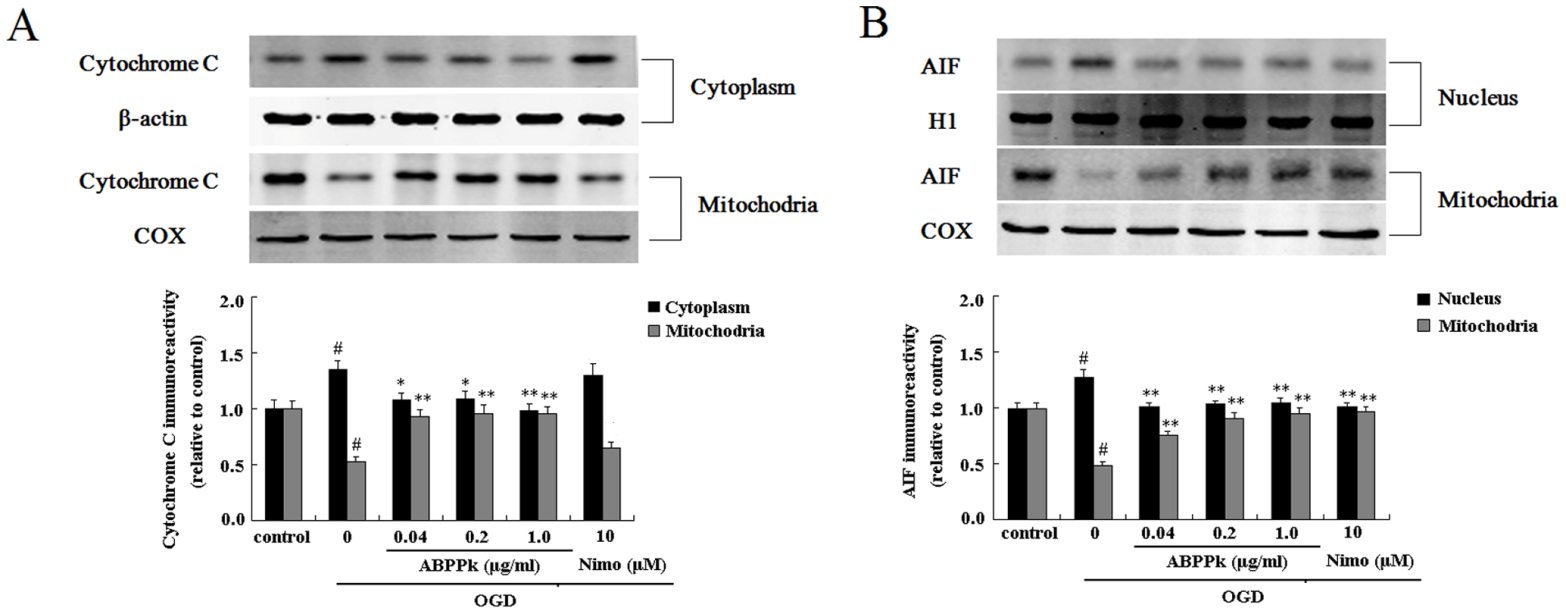
The effects of ABPPk on release of cytochrome c and AIF in OGD model. Representative Western blot image (the upper panel) and the resulting histogram (the lower panel) showing the cytochrome c expression in the cytoplasm and mitochodria (A) and the AIF expression in the nucleus and mitochodria (B) of primary cortical neurons without any treatment (control), exposed to OGD alone, exposed to OGD and then treated with 0.04, 0.2, or 1.0 µg/ml of ABPPk or 10 µM nimodipine (Nimo), respectively. ^#^ *P*<0.01 versus control, *P<0.05 and **P<0.01 versus OGD alone. β-actin, COX, and histone H1 served as loading controls, respectively.

During focal cerebral ischemia, both the loss of ΔΨ_M_ and dysregulation of Bcl-2 family members modulate the apoptosis pathway and subsequently lead to the release of pro-apoptotic cytochrome c and apoptosis-inducing factor (AIF) [11]. In this study, Western blot analysis indicated that exposure to OGD induced mitochondrial release of cytochrome c and AIF in primary cortical neurons (Fig. 8). ABPPk treatment, in a dose-dependent manner, suppressed OGD-stimulated release of these 2 cell death mediators. The significant effects of ABPPk were observed at all 3 doses (0.04, 0.2 and 1.0 µg/ml) for both cytochrome c release and AIF release.

### ABPPk inhibited reactive oxygen species (ROS) production in primary cortical neurons exposed to OGD

**Figure 9:**
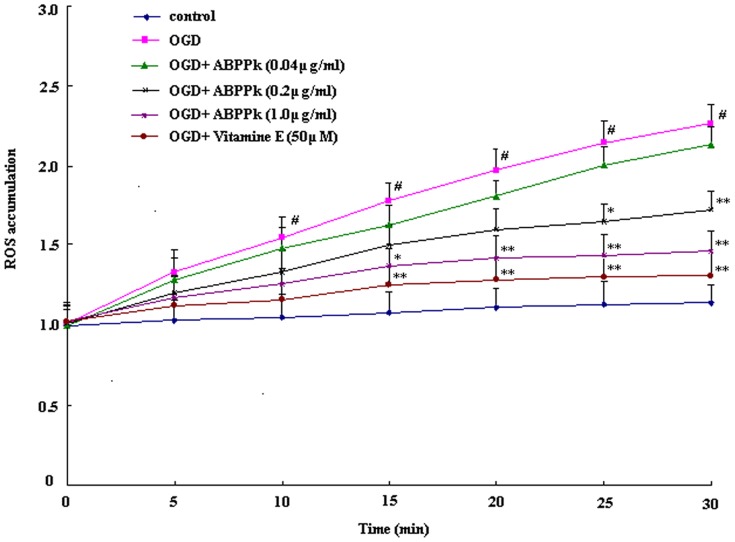
The effects of ABPPk on ROS production in OGD model. Curve lines showing ROS production, as measured by DCFH-DA staining, from primary cortical neurons without any treatment (control), exposed to OGD alone, exposed to OGD and then treated with 0.04, 0.2, or 1.0 µg/ml of ABPPk or 50 µM vitamine E, respectively. ^#^ *P*<0.01 versus control, *P<0.05 and **P<0.01 versus OGD alone.

Molecular probe, 2′,7′-dichlorofluorescin diacetate (DCFH-DA), was used to monitor intracellular ROS levels in a microplate fluorometer [13]. ROS production was measured immediately in OGD-injured primary cortical neurons at 0, 5, 10, 15, 20, 25, 30 min after OGD in the presence or absence of 3 doses of ABPPk or 50 µM vitamin E. Exposure of cortical neurons to OGD insult caused elevation of ROS production. ABPPk suppressed OGD-induced elevation in ROS production, showing a dose-dependent pattern (Fig. 9).

## Discussion

ABPPk, an active HPLC fraction from ABPP, was investigated for its potential neuroprotective action in experimental models of cerebral ischemia designed both under *in vivo* and *in vitro* conditions. ABPPk itself caused no deleterious effects on primary cortical neurons, which may be taken as a prerequisite for examining the protective effect of ABPPk. After exposure of primary cortical neurons to OGD (an *in vitro* model used to mimic cerebral ischemia), MTT assay as well as morphological observation showed that ABPPk rescued OGD-induced cell viability loss in primary cortical neurons. Similarly, intravenous administration of ABPPk to MCAO rats (an *in vivo* model used to mimic cerebral ischemia) reduced the mortality of rats and improved neurological deficit parameters in a dose-dependent manner.

Although the core of infarct zone exposed to the most dramatic blood flow reduction undergoes necrotic cell death, many neurons in the ischemic penumbra or peri-infarct zone may undergo apoptosis at a considerable period after stroke, and thus they are potentially recoverable via post-stroke therapy [14], [15]. To test the effects of ABPPk on the nuclear changes associated with apoptosis, Hoechst and TUNEL staining were performed. The results indicated that ABPPk was likely to attenuate OGD- or MCAO-induced neuronal apoptosis, or contribute to the delay of neuronal death. We also measured the expression of cleaved caspase-3 (17 kD) because it is the ultimate executioner caspase causing the apoptosis-related nuclear changes [16]. We noted that ABPPk inhibited activation and cleavage of caspase-3 in OGD and MCAO models.

Mitochondria are membrane-bound organelles that oxidize complex metabolic fuel. It has long been thought that mitochondrial dysfunction plays a pivotal role in the pathogenesis of various neurological disorders, including stroke [17]–[19]. Accordingly, mitochondria are important in regulating both caspase-dependent and -independent apoptotic pathways [20]. Mitochondrial alterations, in particular the collapse of ΔΨ_M_, is believed to be an early event in the apoptotic process and to affect mitochondrial membrane permeability and release of cytochrome c and AIF [21]–[23]. To determine the effect of ABPPk on ΔΨ_M_, the membrane-permeant JC-1 dye was used to monitor the change in ΔΨ_M_ [24]. JC-1 exhibits ΔΨ_M_-dependent accumulation in mitochondria, as indicated by a fluorescence emission shift from green (JC-1 monomer fluorescence, em = 525 nm) to red (J-aggregate fluorescence, em = 590 nm). The green fluorescence (monomeric form) represents a lower ΔΨ_M_, whereas the red fluorescence (aggregate form) represents a high ΔΨ_M_. ΔΨ_M_ can be expressed as the intensity ratio of red fluorescence to green fluorescence. A reduction in the intensity ratio (ΔΨ_M_) represents mitochondrial depolarization. Confocal microscopy and flow cytometry of JC-1 staining indicated that ABPPk recovered OGD-induced mitochondrial depolarization, suggesting that the neuroprotective effect of ABPPk might be linked to the recovery of ΔΨ_M_.

Apoptosis-related gene family plays a role in intracellular apoptotic signal transduction by regulating the mitochondrial membrane permeability [25], [26]. Anti-apoptotic family members (e.g. Bcl-2 and Bcl-xL) inhibit apoptosis by preventing mitochondrial depolarization [27], whereas pro-apoptotic family members (e.g. Bax) promote apoptosis by inducing mitochondrial depolarization and cytochrome c release [28]. In this study, ABPPk increased the expression of Bcl-2 and Bcl-xL and decreased the expression of Bax in primary cortical neurons exposed to OGD. The results, together with the aforementioned observations about mitochondrial changes, suggested that ABPPk-induced recovery of mitochondrial dysfunction might be correlated to its modulation of apoptosis-related gene expression.

The essential step in the mitochondria-dependent apoptosis pathway is the formation of the apoptosome, a multimeric protein complex consisting of cytochrome c, apoptotic protease activating factor-1 (Apaf-1), pro-caspase 9, and adenosine triphosphate (ATP) [29]. Cytosolic cytochrome c mediates the oligomerization of Apaf-1 and the formation of the apoptosome complex, which cleave the initiator caspase 9, thereby activating the terminal caspases such as caspase-3, -6, and -7 [30]. At this point, release of cytochrome c from mitochondria is vital for the formation of the apoptosome. AIF is a putative caspase-independent effector to regulate the mitochondrial membrane permeability upon apoptosis [31]. Normally AIF is found behind the outer membrane of the mitochondrion. When the mitochondrion is damaged, however, AIF moves to the cytosol and translocates to the nucleus, leading to a caspase-independent cell death process, including DNA fragmentation and chromatin condensation [32]. The release of cytochrome c and AIF from mitochondria triggers downstream caspase-3 activation and additional caspase-independent cell death events and likely plays an important role in mediating neuronal death after cerebral ischemia [33], [34]. Blocking the release of apoptogenic factors into the cytoplasm could prevent cell death [35], [36]. In this study, ABPPk abrogated the release of cytochrome c and AIF from mitochondria in OGD-injured primary cortical neurons, suggesting that the neuroprotective effect of ABPPk might be mediated by mitochondrial blockade of apoptogenic factors (cytochrome c and AIF).

Although the basic function of mitochondria is to generate ATP by oxidative phosphorylation through the electron transport chain, overproduction of ROS by mitochondria is involved in ischemic injury [37], [38], which is caused not only by OGD-induced loss in energy supply but also by oxidative stress [39]. Mitochondria are both targets and sources of oxidative stress [40]. ROS production is involved in the pathogenesis of acute brain injury, and becomes an important therapeutic target [41], [42]. In this study, ABPPk inhibited OGD-induced ROS production, suggesting that the neuroprotective effect of ABPPk might be associated with alleviation of intracellular ROS generation.

To summarize, ABPPk protected primary cortical neurons or rat brain against OGD or MCAO-induced cell or tissue damage, and ABPPk inhibition of neuronal apoptosis might be mediated by mitochondrial-dependent pathways, including modulation of apoptosis-related gene expression, regulation of mitochondrial dysfunction through restoring mitochondrial membrane potential, reducing release of mitochondrial apoptogenic factors, and inhibiting intracellular ROS production. The neuroprotective effect of ABPPk may suggest the possible use of ABPPk in the treatment and prevention of cerebral ischemic insult.

## Materials and Methods

### Materials

TUNEL assay kit was purchased from Promega (Madison, WI). MTT, TTC, JC-1, DCFH-DA, Hoechst 33342, rabbit polyclonal antibody against Bax, Bcl-2, Bcl-xL, AIF, or histone H1, mouse monoclonal antibody against β-Tubulin III, or β-actin were purchased from Sigma (St. Louis, MO). Mouse monoclonal antibody against Cytochrome C was purchased from BD Pharmingen (San Diego, CA). Rabbit polyclonal antibody against cleaved caspase-3 (Asp175) was purchased from Cell signaling Technology (Danvers, MA). Rabbit polyclonal antibody against caspase-3 and mouse monoclonal antibody against COX IV was purchased from Abcam (Cambridge, Mass., UK). IRDye 800 conjugated goat anti-mouse IgG or donkey anti-rabbit IgG was purchased from Rockland (Gilbertsville, PA). Mammalian protein extraction reagent (M-PER), tissue protein extraction reagent (T-PER), nuclear and cytoplasmic extraction reagents (NE-PER), mitochondria isolation kit and Coomassie plus Bradford assay kit were purchased from Thermo Scientific Pierce (Rockford, IL). All standard culture reagents were obtained from Gibco (Grand Island, NY).

### HPLC separation of ABPP

The root of *Achyranthes bidentata* Blume was purchased from a local Chinese medicine grocery and identified by Professor Haoru Zhao from China Pharmaceutical University. ABPP was extracted from *Achyranthes bidentata* Blume as previously described. For preparation of ABPPk, HPLC was performed with a Waters System (Waters, Milford, MA) consisting of Waters Alliance e2695 and Waters 2996 Photodiode Array Detector. A C18 reverse phase-performance liquid chromatography column (4.6 mm×250 mm, 5 µm i.d. Waters, Milford, MA) was applied, and a linear gradient elution was performed with 0.1% trifluoroacetic acid in water/acetonitrile (water ratio, 80∼47% by volume) at a flow rate of 1.0 ml/min. For characterization, the eluted 12 fractions, referred to as ABPPa – ABPPl, were monitored by UV spectrophotometry at 220 nm. They were centrifuged and concentrated in a vacuum freeze drying machine to yield powders, which could be easily dissolved in pure water, saline, or culture medium at a desired concentration for use.

### Cell culture and treatment

Primary culture of cortical neurons was prepared from embryonic day 18 (E 18) Sprague-Dawley (SD) rats as described previously with minor modifications [43]. In brief, animals were sacrificed by cervical dislocation under anesthesia, and the brain was quickly removed. The cortex was dissected out on a cold stage and treated with 0.25% trypsin at 37°C for 10 min. The cortex cells were suspended in DMEM medium containing 10% fetal bovine serum (FBS), and plated onto poly-lysine coated plates at a density of 1 or 2×10^5^ cells/cm^2^ to allow incubation at 37°C in a humidified atmosphere of 95% air and 5% CO_2_ for 4 h. After cells attached to the substrate, the culture medium was replaced with neuronal culture medium (serum-free Neurobasal medium with 2% B27 supplement, 0.5 mM glutamine) for 5 d incubation, and cytosine arabinoside (1 µM) was then added to prevent glial cell proliferation. The cells were characterized by immunocytochemistry with anti-β-Tubulin III, indicating that primary culture contained about 93% neurons.

Primary cortical neurons were subjected to OGD insult as described previously [44], [45]. In brief, the cells were transferred to the glucose-free DMEM bubbled with 95% N_2_/5% CO_2_ in a sealed humidified modular incubator chamber (MIC-101, Billups-Rothenberg, Del Mar, CA) for 6 h incubation. For reperfusion, the exposure medium was replaced with plain neuronal culture medium, or with neuronal culture medium added with ABPPk (0.04, 0.2, or 1.0 µg/ml) or nimodipine (10 µM), respectively, and cell culture was placed in a normoxic incubator for another 24 h incubation. At the end of cell treatments, cell culture was subjected to various assessments. The cells cultured in plain DMEM and then plain neuronal culture medium both with ambient oxygen for 6 and 24 h, respectively, served as control (no exposure to OGD).

### Cell viability assay

MTT assay was used to determine cell viability of primary cortical neurons. The MTT solution was added to the cell sample to make final concentration of 0.5 mg/ml, and the reaction mixture was allowed to incubate at 37°C for 4 h before addition of 20% sodium dodecyl sulfide (SDS) to dissolve the resulting formazan. The absorbance (OD) value was measured by spectrophotometry at 570 nm with an EIX-800 Microelisa reader (Bio-Tek Inc., USA). The cell viability was expressed as a percentage of control value.

### Animals and surgical procedures

Male SD rats, weighing 200±10 g, were provided by the Experimental Animal Center of Nantong University, Nantong, China. All experimental procedures involving animals were conducted as per institutional animal care guidelines of Nantong University and were approved ethically by the Jiangsu administration committee for experimental animals, China.

Surgery was performed as described previously [4]. After animals were anesthetized, a monofilament nylon suture with PLL-coated distal end (Sunbio Biotech, Beijing, China) was inserted into the internal carotid artery to cause transient MCAO. At 2 h after MCAO, reperfusion was induced by filament withdrawal, and at the start of reperfusion 1 ml of ABPPk (0.1, 0.2, or 1.0 mg/kg), nimodipine (1.0 mg/kg), or saline (vehicle) was intravenously (iv) injected into the femoral vein of animals, respectively (n = 24 per group). Sham-MCAO animals (n = 12) were subjected to the same surgical procedure except that a monofilament suture was not advanced beyond the internal carotid bifurcation. During the whole surgical period, the body temperature of animals was maintained at 36.5 ± 0.5°C by the use of a heating pad controlled by a rectal probe.

### Neurological deficit evaluation

After 2 h of MCAO and 24 h of reperfusion, the modified neurological severity scores (mNSS) were measured by an investigator who was blinded for treatment assignment. The mNSS consists of motor, sensory, reflex, and balance tests, and the items are scored on an 18-point scale (normal score, 0; maximal deficit score, 18). The higher is the score, the more severe is the injury [46].

The brain infarct size was measured by riphenyltetrazolium chloride (TTC) staining as described previously with modifications [47]. In brief, after 2 h of MCAO and 24 h of reperfusion, animals were sacrificed under deep anesthesia, and the brain was quickly dissected and cut into 2-mm-thick coronal sections using a rat brain matrix (Sunny Instruments, Beijing, China). The slices were immediately placed in PBS containing 0.5% TTC at 37°C for 20 min, and then fixed in buffered 4% formaldehyde solution for 1 h at room temperature, followed by image capture with a video camera (Canon PowerShot S60; Canon, Tokyo, Japan), and analysis of the infarct volumes by the Q550 IW image analysis system (Leica Imaging System Ltd., Cambridge, UK). The infarct volume was calculated as [(V_c_−V_l_)/V_c_]×100%, where V_c_ is the volume of control hemisphere and V_l_ is the volume of noninfaracted tissue in the lesioned hemisphere.

### Hoechst staining

Primary cortical neurons were fixed in 4% paraformaldehyde at room temperature for 20 min, and then stained with 5 µg/ml Hoechst 33342 dye for 10 min, followed by observation under DMR fluorescence microscopy (Leica Microsystems, Wetzlar, Germany) with fluorescence excitation at 340 nm and emission at 510 nm. The cells with fragmented or condensed DNA were counted as apoptotic cells, and the ratio of apoptotic cells to total cells was calculated.

### TUNEL assay

Rats were transcardially perfused, and the brain was harvested. The procured samples were post-fixed in buffered 4% paraformaldehyde, dehydrated in a graded sucrose series, and then cut on a cryostat into coronal sections at a thickness of 20 µm. TUNEL procedures were applied to frozen brain sections according to the kit protocols. In brief, the sections were fixed in 4% methanol-free formaldehyde, permeabilized in 0.2% Triton X-100, and covered with equilibration buffer. DNA strand breaks were labeled with fluorescein-12-dUTP in an equilibration buffer added with recombinant TdT, avoiding exposure to light. The negative control was incubated with an incubation buffer without recombinant TdT. The reaction was terminated with 2×SSC (300 mM sodium chloride and 30 mM sodium citrate, pH 7.4). Afterwards, the sections were stained with 1 µg/ml propidium iodide (PI) at room temperature in the dark. Apoptotic (TUNEL-positive) cells were detected as localized bright green cells in a red background under scanning laser confocal microscopy (Leica, Heidelberg, Germany). Data are expressed as the ratio of apoptotic cells to total cells.

### Measurement of mitochondrial membrane potential (▵Ψm)

The fluorescent probe JC-1 was used to measure the ▵Ψm of primary cortical neurons. For fluorescence images, culture slides were incubated with 2 µM JC-1 at 37°C for 15 min. Slides were washed and mounted, and fluorescence images were then acquired in a fluorescence microscope (Leica, Wetzlar, Germany)

For flow cytometry, primary cortical neurons (1×10^6^) were collected by trypsinisation, washed in PBS and incubated with 2 µM JC-1 at 37°C for 15 min. Cells were washed, and analyzed by flow cytometer (BD FACScalibur, BD Bioscience, San Jose, CA). Photomultiplier settings were adjusted to detect JC-1 monomer fluorescence signals on the FL1 detector (green fluorescence, centered at ∼390 nm) and JC-1 aggregate fluorescence signals on the FL2 detector (red fluorescence, centered at ∼340 nm). The intensity ratio of aggregate to monomer (red/green) fluorescence was used for data presentation.

### Western blot analysis

The M-PER, T-PER, NE-PER and mitochondria isolation kit were used for total protein extraction from primary cortical neurons, brain samples, nucleoprotein, and mitochondrial protein. The protein samples were subjected to protein quantification with a Coomassie plus Bradford assay kit.

Identical amounts of protein samples (40 µg per sample) were separated by SDS-PAGE, blotted onto a PVDF membrane, and incubated at 4°C overnight with anti-Bax (1∶2000), anti-Bcl-2 (1∶1000), anti-Bcl-xL (1∶1000), anti-cleaved caspase-3 (1∶1000), anti-caspase-3 (1∶500), anti-AIF (1∶1000), anti-cytochrome C (1∶500), anti-Histone H1 (1∶1000), anti-COX IV (1∶1000), anti-β-actin (1∶2000), respectively. Then, IRDye 800-conjugated affinity purified goat anti-mouse IgG (1∶5000) or donkey anti-rabbit IgG (1∶5000) was applied at room temperature. The images were scanned with Odyssey infrared imaging system (LI-COR), and the data were analyzed with PDQuest 7.2.0 software (Bio-Rad). The data were expressed as the values relative to the control value.

### Measurement of intracellular ROS

Intracellular accumulation of ROS was measured with DCFH-DA as described previously [4], [48], [49] with minor modifications. To test the effects of ABPPk on the OGD-induced generation of ROS, cells were washed twice with Ca^2+^, Mg^2+^-free HBSS, then incubated with 20 µM DCFH-DA in Ca^2+^, Mg^2+^-free HBSS for 30 min at 37°C in the dark and washed twice with Ca^2+^, Mg^2+^-free HBSS. The change of ROS was estimated by the fluorescence ratio of the dye-loaded neurons after OGD stimulation immediately and 5, 10, 15, 20, 25, 30 min during reperfusion period. The dye-loaded cells were measured for fluorescence with a fluorescence microplate reader (Bio-Tek Inc., USA; excitation, 485 nm; emission, 535 nm).

### Statistical analysis

All data are presented as means±SEM for 3 separate experiments (each in triplicate or duplicate). Statistical analysis was performed by one-way analysis of variance (ANOVA) and subsequent Bartlett's test. Differences were considered statistically significant at *P*<0.05.
